# Quality of tumor lysates used for pulsing dendritic cells is influenced by the method used to harvest adherent tumor cells

**DOI:** 10.1186/1756-0500-4-153

**Published:** 2011-05-26

**Authors:** Gloria Isabelle Herzog, Ghasem Solgi, Denis S Wiegmann, Christian Nienhaus, Hubert Schrezenmeier, Tatjana Yildiz, Ramin Lotfi

**Affiliations:** 1 Institute of Transfusion Medicine, University of Ulm, Germany; 2 Institute of Clinical Transfusion Medicine and Immunogenetics Ulm, German Red Cross Blood Services Baden-Württemberg-Hessen, Helmholtzstr. 10, 89081 Ulm, Germany; 3 Immunology department, School of Medicine, Ahvaz Jundishapur University of Medical Sciences (AJUMS), Iran

## Abstract

**Background:**

Lysates from tumor cells are reported to induce maturation of dendritic cells (DCs) and are used in clinical settings for DC-based vaccination against solid tumors. Nevertheless, the maturation inducing effect of tumor lysates on DCs is discussed controversially and the efficacy of tumor vaccines varies significantly.

**Findings:**

Using three individual adherent colorectal tumor cell lines we also faced the difficulty to obtain consistent results regarding maturation inducing effect of tumor lysates on DCs. Therefore, we compared different methods to prepare tumor cell lysate and could demonstrate that trypsinizing as a method to harvest adherent tumor cells has a significant negative impact on biologic activity of tumor lysates. Specifically, we assessed induction of maturation markers CD40, CD80, and CD86 on DCs which were treated with differently prepared lysates.

**Conclusions:**

Trypsinizing is a very common way of harvesting adherent cells from culture flasks. Our results shall call investigators' attention to the enzymatic activity of trypsin degrading some possibly important proteins on the surface of cultured cells. Specifically for DC-based vaccination against tumor antigens investigators should avoid trypsin.

## Findings

Lysates from tumor cells are reported to induce maturation of dendritic cells (DCs) and are used as a source for tumor specific antigens [1,2], but as the maturation inducing effect is not strong enough [3], cytokine cocktails are added to DC cultures which are pulsed with tumor lysate [3,4], in the clinical setting of DC-based tumor vaccination. We aimed to enhance the effect of tumor lysate with regard to induction of DC maturation, but as reported by others [3] we had to face the problem that the tumor lysate rather did not induce DC maturation, in terms of upregulation of surface markers CD40, CD80, and CD86. We aimed to clarify the reason for conflicting results concerning lysate-induced DC maturation.

Based on its protease activity trypsin cleaves cell surface proteins necessary for adherence. Trypsin at concentrations between 0.25% and 0.5% is generally used to detach and to harvest adherent (tumor) cells at the cost of loss of a portion of surface antigens. Therefore, we hypothesized that trypsin may crucially impact antigenicity of tumor lysate and subsequently its biological activity.

By building a stable chelate with calcium ions ethylenediaminetetraacetic acid (EDTA) deprives cells from calcium which is crucial for cell adherence [5]. EDTA can substitute for trypsin to detach adherent cells without interfering with expressed surface antigens.

## Results and Discussion

### Lysates from tumor cells harvested by trypsin do not induce expression of maturation markers on DCs

**Figure 1:**
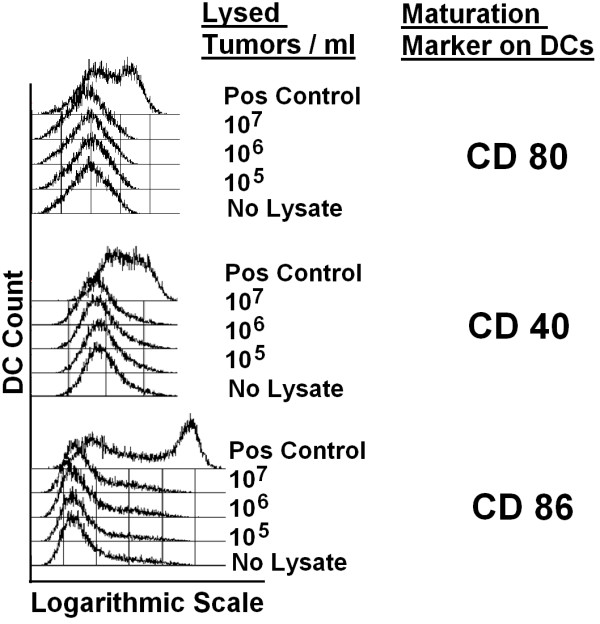
**Tumor lysates from trypsinized tumor cells do not induce maturation of DCs**. Adherent tumor cells were harvested by using 0.25% trypsin to detach the cells, washed and lysed in PBS by repeated freezing and thawing. Human monocyte-derived immature DCs were stimulated for 3 days with supernatants of indicated concentrations of lysed tumor cells. 12 individual experiments with lysates from 3 different cell lines (HCT-116, CACO-2, COLO-678) were performed and results with lysates from HCT-116 cells are shown as representative example.

iDCs were stimulated for threee days with supernatants of lysed tumor cells which have been harvested by using trypsin, DCs which have been stimulated this way failed to express maturation markers CD 40, CD80, or CD86 (Figure 1). Same results were obtained in several experiments with lysates from three individual colorectal tumor cell lines. Results from lysed HCT-116 cells are shown as representative example.

### Lysates from tumor cells can induce expression of maturation markers on DCs if trypsin is replaced by EDTA

**Figure 2:**
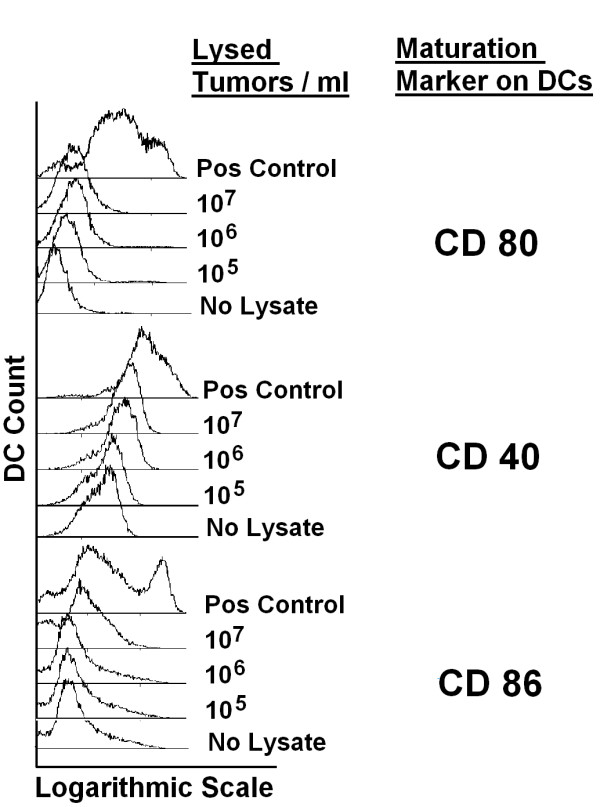
**Tumor lysates from cells which have been harvested using EDTA induce DC maturation in a dose dependent manner**. HCT-116 colorectal tumor cells were harvested by using 0.02% EDTA to detach the cells, washed and lysed in PBS by repeated freezing and thawing. Human monocyte-derived immature DCs were stimulated for 3 days with supernatants of indicated concentrations of lysed tumor cells. Shown are representative results from 6 individual experiments with lysates from 2 different cell lines (HCT-116, CACO-2).

Due to its non-specific protease activity trypsin may not only degrade proteins associated with cell adhesion but also those cell surface proteins which are crucial for recognition of tumor cells and induction of DC maturation, thus we substituted for trypsin by using EDTA. In contrast to lysates from trypsinized cells, lysates from tumor cells which have been harvested by using EDTA (Figure 2) or a scraper (data not shown) induced the upregulation of maturation markers CD40, CD80, and CD86 on the surface of DCs after three days of stimulation.

**Figure 3:**
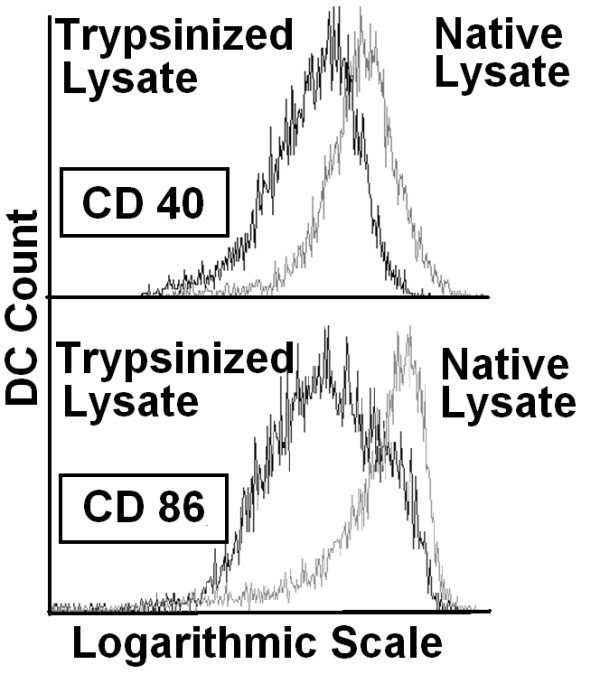
**Trypsin abolishes maturation inducing effect of tumor lysate**. Lysate from HCT-116 colorectal tumor cells which have been harvested by using 0.02% EDTA was were divided, one part was exposed to trypsin (0.25%) and the other part was sham treated with PBS for 5 min. In order to inactivate trypsin, 100% human AB serum (2× higher volume) was added to both arms after 5 minutes. DCs were stimulated for 3 days with trypsin-exposed or sham-treated tumor lysate and examined for expression of surface maturation markers CD40 and CD86.

In order to convince ourselves that the maturation inducing effect of tumor cell lysate is based on proteins which are degraded by trypsin and not due other factors found within tumor lysate we first exposed the lysate from HCT-116 tumor cells (harvested by using just EDTA) to trypsin (0.25% for 5 min) and used it afterwards to stimulate DCs for 3 days, results were compared to sham-treated lysates (exposed to PBS). Flow cytometric analysis of surface maturation markers on DCs shows abolished effect of trypsin-exposed lysate (Figure 3).

Trypsinizing is a very common way of harvesting adherent cells from culture flasks. Our results shall call investigators' attention to the enzymatic activity of trypsin degrading not only proteins associated with cell adherence but also those cell surface proteins which are crucial for recognition of tumor cells and induction of specific immune response. Specifically for DC-based vaccination against tumor antigens investigators should avoid trypsin. According to our results, we substituted for trypsin with EDTA when harvesting DCs which were supposed to be analyzed for expression of surface maturation markers by FACS, appreciating the proteolytic effect of trypsin some research group alternatively pre-incubate their DCs with ice-cold PBS for 30 to 60 minutes, making it possible to harvest the DCs without the need for further chemical treatment.

## Methods

### Standard cell culture conditions

As standard culture media we used phenol-red free DMEM (Gibco) supplemented with 10% human AB serum (German Red Cross Blood Services Baden-Württemberg-Hessen) and containing 100 U/ml penicillin, 100 μg/ml streptomycin (Gibco). All cells (tumor cells, monocytes, and DCs) were cultured in this media and incubated in a humidified atmosphere at 37°C with 5% CO2.

### Generation of tumor cell lysates

Three colorectal tumor cell lines HCT-116, CACO-2, COLO-678 (all from DSMZ, Germany) were cultured in our standard media. Cells were splitted for further passages or harvested to generated lysates once they reached a confluency of about 90%.

Tumor cells were detached by incubating the cells at room temperature in 0.25% Trypsin (Gibco) in 0.02% EDTA (trypsin/EDTA Sigma-Aldrich) or alternatively in 0.02% EDTA alone for 5 and 15 min, respectively. In order to inactivate trypsin/EDTA, two parts of standard cell culture media [containing 10% serum] per one part of used trypsin/EDTA was added to the cell suspension. Detached cells were washed with PBS and resuspended in sterile PBS (Lonza) at a concentration of about 1 × 10^8^ cells/ml and lysed by 5 cycles of freeze-thawing (F/T) (-80 to 37°C). The viability following treatment was assessed using trypan blue exclusion and was always below 0.1%. Lysates were spun down hard (16,300 × *g*) twice and the soluble supernatant was used at given concentrations.

In some experiments obtained tumor cell lysates were divided, one part was exposed to indicated concentrations of trypsin and the other part was sham treated with PBS. In order to inactivate trypsin, 100% human AB serum (2× higher volume) was added to both arms after 5 minutes. The activity of trypsin-exposed lysates was compared to its sham-treated (PBS-exposed) counterpart.

### Isolation of peripheral blood mononuclear cells and purification of monocytes and autologous lymphocytes

With the permission and supervision of the Institutional Review Board (Ethical Committee) of the University of Ulm human peripheral blood mononuclear cells were purified from whole blood by density gradient centrifugation using Biocoll (Biochrom AG, Germany), monocytes were positively selected using anti-CD14 antibodies labelled with magnetic beads (Miltenyi Biotec) following the manufacturer's instructions. The purity was assessed under microscope and was at least 95%.

### Generation of monocyte derived human DCs

Immature DCs (iDCs) were generated from human monocytes within 5 days in the presence of IL-4 (500 U/ml) and GM-CSF (1000 U/ml) [6] (both cytokines from R&D Systems). iDCs were harvested by using EDTA, resuspended in our standard media and transferred into a flat bottom 24 well plate (Nunc) at a density of 3 to 5 × 10^5^ iDCs/well and ml. iDCs were stimulated with indicated concentrations of individual tumor cell lysates for 3 further days and harvested then by using EDTA to detach the DCs. The mixture of IFNγ (100 ng/ml), TNFα (50 ng/ml), IL1β (10 ng/ml) (all from R&D Systems), and LPS (100 ng/ml) (Sigma-Aldrich) served as positive control for induction of DC maturation.

### Cell surface staining and flow cytometry

DCs were fixed in 2% paraformaldehyde (Fluka) for 20 min at room temperature, washed with PBS. Non-specific binding sites on DCs were blocked for at least 10 min with mouse whole IgG (Jackson ImmunoResearch Laboratories). Specific staining was performed for at least 1 h at room temperature or alternatively overnight at 4°C using fluorescently labelled murine anti-human CD40, CD80 and CD86 or respective isotype controls (R&D Systems). The fluorescence of stained cells was assessed using BD FACScan (BD Biosciences). At least 20,000 events were acquired in all flow cytometric analyses.

## Competing interests

The authors declare that they have no competing interests.

## Authors' contributions

GIH and RL designed the study and wrote the manuscript. GIH and GS performed DC maturation assays using both lysates from tumor cells harvested using EDTA and trypsin. DW performed dose-response experiments with lysates from tumor cells harvested by using EDTA. CN performed dose-response experiments with lysates from tumor cells harvested by using trypsin. TY performed dose-response experiments comparing the effect of lysates from tumor cells harvested by using EDTA or trypsin. HS provided material and cells and participated in study's design and coordination and helped to draft the manuscript. All authors read and approved the final manuscript.
